# Using [^11^C]Ro15 4513 PET to characterise GABA-benzodiazepine receptors in opiate addiction: Similarities and differences with alcoholism

**DOI:** 10.1016/j.neuroimage.2016.02.005

**Published:** 2016-05-15

**Authors:** Anne Lingford-Hughes, James Myers, Ben Watson, Alastair G. Reid, Nicola Kalk, Adrian Feeney, Alexander Hammers, Daniela A. Riaño-Barros, Colm J. McGinnity, Lindsay G. Taylor, Lula Rosso, David J. Brooks, Federico Turkheimer, David J. Nutt

**Affiliations:** a Centre for Neuropsychopharmacology, Imperial College London, Du Cane Rd., London W12 0NN, United Kingdom; b Psychopharmacology Unit, University of Bristol, Whitson Street, Bristol BS1 3NY, United Kingdom; c MRC Clinical Sciences Centre, Faculty of Medicine, Imperial College, London, United Kingdom; d Hammersmith Imanet Ltd., Hammersmith Hospital, Du Cane Rd., London W12 0NN, United Kingdom

## Abstract

The importance of the GABA-benzodiazepine receptor complex and its subtypes are increasingly recognised in addiction. Using the α1/α5 benzodiazepine receptor PET radioligand [^11^C]Ro15 4513, we previously showed reduced binding in the nucleus accumbens and hippocampus in abstinent alcohol dependence. We proposed that reduced [^11^C]Ro15 4513 binding in the nucleus accumbens was a marker of addiction whilst the reduction in hippocampus and positive relationship with memory was a consequence of chronic alcohol abuse. To examine this further we assessed [^11^C]Ro15 4513 binding in another addiction, opiate dependence, and used spectral analysis to estimate contributions of α1 and α5 subtypes to [^11^C]Ro15 4513 binding in opiate and previously acquired alcohol-dependent groups. Opiate substitute maintained opiate-dependent men (*n* = 12) underwent an [^11^C]Ro15 4513 PET scan and compared with matched healthy controls (*n* = 13). We found a significant reduction in [^11^C]Ro15 4513 binding in the nucleus accumbens in the opiate-dependent compared with the healthy control group. There was no relationship between [^11^C]Ro15 4513 binding in the hippocampus with memory. We found that reduced [^11^C]Ro15 4513 binding was associated with reduced α5 but not α1 subtypes in the opiate-dependent group. This was also seen in an alcohol-dependent group where an association between memory performance and [^11^C]Ro15 4513 binding was primarily driven by α5 and not α1 subtype. We suggest that reduced α5 levels in the nucleus accumbens are associated with addiction since we have now shown this in dependence to two pharmacologically different substances, alcohol and opiates.

## Highlights

• Lower [^11^C]Ro15 4513 binding is evident in the nucleus accumbens of opiate addicts.
• This appears primarily due to lower levels of α5 subtype of the GABA-A receptor.
• Lower [^11^C]Ro15 4513 binding is similarly found in alcoholism.
• Lower levels of α5 GABA-A receptor in nucleus accumbens may underpin addiction.

## Introduction

Opiate addiction and its treatment is a global health issue. In the US, 1.8% of individuals over the age of 12 have tried heroin in their lifetime, with 0.1% in the past month (National Survey of Drug Use and Health, 2012). In England, there are an estimated ~ 300,000 opiate and/or crack users (8.67 per 1000 of 15 to 64 year olds; Statistics on Drug Misuse: England, 2013). Many addicts relapse or find it hard to achieve abstinence, with only about 50% leaving treatment drug-free (Statistics on Drug Misuse: England, 2013). To improve understanding and treatment of opiate addiction, characterising its underlying neurobiology is critical. Although heroin and other opioid agonists act on opioid receptors, preclinical evidence supports involvement of the GABA system in their effects (Zetterström and Fillenz, 1990, Finlay et al., 1992 and Creed et al., 2014). Endorphins or mu opiate agonists inhibit GABA-ergic tonic inhibition of dopamine neuronal activity in the ventral tegmental area (VTA) resulting in increased dopamine neuronal firing and may thus contribute to the desire or compulsion to use opiates (Johnson and North, 1992 and Ting-A-Kee and van der Kooy, 2012).

There are multiple subtypes of the GABA-benzodiazepine receptor with the type of α subunit determining benzodiazepine and alcohol sensitivity (Rudolph and Mohler, 2004). There is evidence that the α1, α2 and α5 subtypes contribute to drug-liking and/or drug-seeking in a variety of preclinical models with a range of substances of abuse (Rudolph and Knoflach, 2011, Engin et al., 2012, Dixon et al., 2010, June et al., 2001). Associations have also been reported between variants of the GABA-benzodiazepine receptor gene and alcohol and drug use disorders (Engin et al., 2012, Li et al., 2014). In addition, the α5 subtype plays a role in learning and memory and its impairment under the influence of alcohol (Nutt et al., 2007, Atack, 2011).

[^11^C]Ro15 4513 has a tenfold higher affinity at α5 than α1 receptors (Lingford-Hughes et al., 2002). We have used [^11^C]Ro15 4513 to characterise benzodiazepine receptor binding in man, particularly the α5 subtype, but due to the ubiquity of the α1 subtype, [^11^C]Ro15 4513 PET also can detect this subtype (Myers et al., 2012). We previously reported lower levels of [^11^C]Ro15 4513 binding in the nucleus accumbens of abstinent alcoholics compared with controls (Lingford-Hughes et al., 2012). We proposed that since the extrasynaptic α5 subtype provides tonic inhibition, this lower level of receptors would contribute to instability of this critical dopaminergic brain region which in turn could lead to enhanced drug consumption or seeking. We also reported that in alcoholics, although not healthy controls, α5 receptor subtype availability in the hippocampus positively correlated with verbal memory performance.

This paper complements our [^11^C]Ro15 4513 PET study in alcoholism by studying opiate dependence to assess whether lower levels of [^11^C]Ro15 4513 binding are a marker of addiction. We hypothesised that [^11^C]Ro15 4513 binding in opiate dependence would be lower in the nucleus accumbens compared with controls. Since we suggested the lower [^11^C]Ro15 4513 binding in the hippocampus was a consequence of chronic alcohol abuse, we did not expect to see such a reduction in opiate addiction. Subsequent to our study in alcoholism (Lingford-Hughes et al., 2012), we have developed the use of spectral analysis to assess the contribution of α1 vs α5 subtypes to the [^11^C]Ro15 4513 image (Myers et al., 2012). We report here for the first time levels of these subtype in opiate addiction and in alcoholism.

## Materials and methods

### Participants

Opiate-dependent males (DSM-IV) receiving substitute medication (either methadone (*n* = 9; range 10–75 mg daily; mean daily dose 46.7 mg ± 19.7 mg) or buprenorphine (*n* = 3; 2.8 mg, 10 mg, 24 mg daily; mean 12.3 mg ± 10.8 mg)) were recruited from local addiction outpatient treatment services (see Table 1). The control group consisted of 13 men recruited for this study or other studies (Myers et al., 2012, Stokes et al., 2014) to match the opiate-dependent group. They had never met criteria for abuse or dependence (DSM-IV) on alcohol or opiates, although recreational non-dependent use of other illicit drugs (eg cannabis, ecstasy) was permitted.

In the opiate-dependent group, none were or had been dependent on any other drug including alcohol, cannabis, benzodiazepines or stimulants, although occasionally cocaine, crack or amphetamine or cannabis had been used, mostly when they were younger. All were abstinent from alcohol and illicit drugs at the time of the study confirmed with negative urinalysis and breathalyser reading. Duration of opiate use ranged from 9 to 30 years (mean ± SD, 16 ± 6.8) with most smoking and injecting although 4 had never injected. The opiate-dependent subjects were all current tobacco smokers except for one who had stopped 6 weeks previously; in the control group, 2 were smokers.

Individuals with clinical evidence of hepatic, cognitive or neurological impairment or medical disorder were excluded. No individual had a previous or current history of psychosis. None were taking psychotropic drugs, other than opiate substitute medication, except for one opiate-dependent individual on mirtazapine.

### Assessment: clinical

The opiate-dependent group was assessed using a semi-structured questionnaire for all drugs of abuse, including alcohol and with Adjective Checklist (Jasinski, 1997) for their state at the time of the scan. They underwent a similar assessment as in the alcohol-dependent study which is described elsewhere (Lingford-Hughes et al., 2012) and included Beck Depression Inventory (Beck et al., 1961), Spielberger State–Trait Anxiety Inventory (Spielberger et al., 1970) and the Wechsler Memory Scale and logical memory with immediate and 30-min delayed recall of two stories. The controls recruited for other studies did not complete all these tests (*n* = 9; Stokes et al., 2014).

After complete description of the study to the subjects, written informed consent was obtained. The study was approved by the local Ethics Committees and by the UK Administration of Radioactive Substances Advisory Committee.

### [^11^C]Ro15 4513 PET

We followed the same scanning protocol as described in Lingford-Hughes et al. (2002). [^11^C]Ro15 4513 was synthesised by *N*-methylation of the corresponding *N*-desmethyl derivative with [^11^C]iodomethane. The product was purified by reverse phase HPLC and the specific radioactivity was ~ 14,000 MBq/μmol at end of synthesis.

[^11^C]Ro15 4513 (mean ~ 475 MBq) in ~ 2 ml scans of opiate-dependent individuals and all corresponding control subjects were performed on a Siemens ECAT EXACT HR + (CTI/Siemens, model 962; Knoxville, TN, USA) scanner.

Blood sampling was carried out to produce a calibrated, metabolite corrected plasma input function for quantification of distribution volume with spectral analysis (Lingford-Hughes et al., 2012).

### Image processing

PET emission data were corrected for attenuation and scatter using a 10-min transmission scan and reconstructed using Fourier rebinning and 2D filtered backprojection with a 2.0 mm kernel Ramp filter, into 24 dynamic frames (1 × 30, 4 × 15, 4 × 60, 2 × 150, 10 × 300 and 3 × 600 s). The final reconstructed volume had voxel dimensions of 2.094 × 2.094 × 2.42 mm. All subjects also underwent T1-weighted magnetic resonance imaging (MRI) with a Philips 1.5 T Gyroscan Intera scanner (Philips, Best, The Netherlands), to provide anatomical images to aid in region definition. A T1-weighted protocol was used to acquire images viewed by a clinical Consultant Radiologist, and none were reported as having significant atrophy or abnormalities.

Reconstructed [^11^C]Ro15 4513 images were analysed on Sun workstations (Sun Microsystems, Mountain View, CA, USA) using Analyze AVW version 8.1 (Biomedical Imaging Resource, Mayo Clinic, Rochester, MN; Robb and Hanson, 1991), Matlab 6.5 (The MathWorks, Inc., Cambridge, UK) and SPM5 (available via http://www.fil.ion.ucl.ac.uk/spm/).

Arterial blood input functions calibrated against discrete blood samples using a well counter and further corrected for parent fraction and plasma:blood ratio. Weighted summed PET images were used for rigid-body co-registration to anatomical MRI data.

Maximum probability maps defined in standard space (73 ROIs for the earlier study with alcohol-dependent individuals, and 83 ROIs for opiate dependence; Hammers et al., 2003, Gousias et al., 2008) were fitted after transformation into native space. Prior to regional sampling, the goodness-of-fit of each object map to the individual brain was checked visually. The ROI sampling was performed in Analyze 8.1 to obtain the mean radioactivity concentration through all frames for all regions in the atlas, and used to generate time-activity curves (TACs).

### Spectral analysis

Spectral analysis is a basis function technique that uses a set of poly-exponentials convolved with the parent plasma input function to fit tissue TACs with no prior assumption of model order (Cunningham and Jones, 1993, Turkheimer et al., 1994). Although [^11^C]Ro15 4513 is relatively selective for the GABA-benzodiazepine α5 receptor subtype, there is measureable signal from the ubiquitous α1 subtype, which can be partially quantified using band-pass spectral analysis (Myers et al., 2012). [^11^C]Ro15 4513 *V*_T_ can therefore be described as comprising α1, α5 and non-displaceable components, *V*_α1_ + *V*_α__5_ + *V*_ND_. Although it is not possible to directly quantify the contribution of these components to the total signal, spectral analysis has been used to provide estimates by separating *V*_α__5_ on the basis of slow kinetics. One hundred exponential functions were generated, with the decay parameter logarithmically distributed between 0.00006371 and 1 s^− 1^ (Barros et al., 2010) and convolved with the arterial parent plasma input function (*C*_*P*_) in order to generate a set of kinetic basis functions. A non-negative least-squares algorithm was then used to fit this over-complete basis set to the measured data (*C*_PET_):

$$C_{\mathrm{PET}}\left(t\right)=\sum_{i=1}^{100}\phi_{i}e^{-\theta_{i}t}⊗C_{P}\left(t\right)$$

subject to *ϕ*_*i*_ ≥ 0. Thus, an integral of the slow kinetic component of the spectrum has been hypothesised to relate to GABA_A_α5 binding, and *V*_*α*5_ is defined by the limits *a* (0.00006371 s^− 1^) and *b* (0.001 s^− 1^):

$$V_{\alpha 5}=\sum_{i=a}^{b}\frac{\phi_{i}}{\theta_{i}}$$

Specific binding of [^11^C]Ro15 4513 to α1 is approximated by defining the limits 0.0010–0.00040 s^− 1^. Total volume of distribution (*V*_T_) is defined as the integral of all peaks in the spectrum.

### Statistical analysis

Statistical analyses were conducted using GraphPad Prism (version 6 for Windows, GraphPad Software, San Diego, CA, USA, www.graphpad.com). Relationships between [^11^C]Ro15 4513 volume of distributions *V*_T_, *V*_α__1_ and *V*_α__5_ and measures of memory were tested with non-parametric Spearman's ranks correlation coefficients. Repeated-measures two-way ANOVA with ROI and group (alcohol or opiate-dependent vs controls) was used to compare [^11^C]Ro15 4513 distribution volume in dependent individuals with that in controls. Results were corrected using post hoc Šidák correction for multiple comparisons.

## Results

### Opiate dependence: [^11^C]Ro15 4513 *V*_T_

**Fig. 1:**
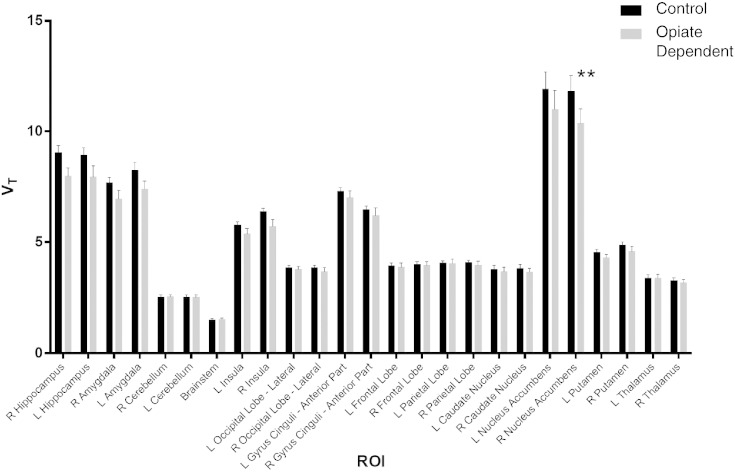
[^11^C]Ro15 4513 *V*_T_ (mean ± SEM) in controls (*n* = 13) and opiate-dependent (*n* = 12) individuals in selected regions of interest (ROIs). There was significantly lower *V*_T_ in the addict group across all ROIs with a two-way ANOVA ((*F*(1, 1743) = 27.85; *p* < 0.0001), the right nucleus accumbens remained significant (*t* = 3.971; *p* < 0.01) after multiple comparison correction (**).

There was a significant effect of group in the two-way ANOVA comparing [^11^C]Ro15 4513 *V*_T_ in all ROIs between opiate-dependent and healthy control groups, with lower *V*_T_ in the opiate-dependent group (*F*(1, 1743) = 27.85; *p* < 0.0001). Data from ROIs selected for their relevance to addiction and [^11^C]Ro15 4513 *V*_T_ binding are shown in Fig. 1. After correction for multiple comparisons, the right nucleus accumbens [^11^C]Ro15 4513 *V*_T_ in the opiate-dependent group was the only ROI where lower *V*_T_ compared with healthy controls remained significant (*t* = 3.971; *p* < 0.01). No difference in nucleus accumbens volume, as defined by our atlas registration, was found between the groups. There was a trend (*p* = 0.06) towards lower *V*_T_ in the hippocampus of opiate-dependent individuals, in contrast to the significantly lower [^11^C]Ro15 4513 *V*_T_ in the alcohol-dependent group (see below).

**Fig. 2:**
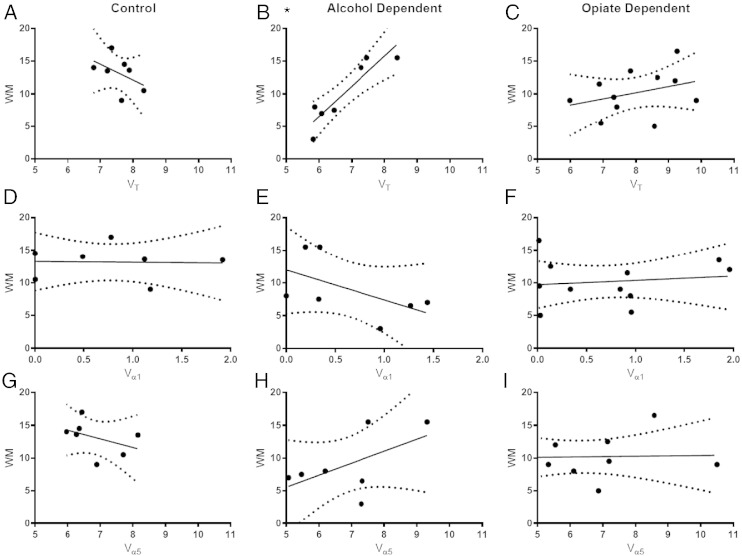
Correlations of [^11^C]Ro15 4513 *V*_T_, *V*_α__1_ and *V*_α__5_ in the hippocampus with Wechsler Fig. 1 delayed verbal memory score (WM; with 95% confidence intervals) in controls (*n* = 11; A, D, F), alcohol-dependent (*n* = 8; B*, E, H; see Supplementary information for more details) and opiate-dependent (*n* = 12; C, F, I) individuals. (*p < 0.005).

No significant correlations were found between delayed verbal memory performance and [^11^C]Ro15 4513 *V*_T_ in opiate-dependent individuals in the hippocampus (see Fig. 2C). There was no significant association of age or any clinical variables with *V*_T_. We did not see differences in *V*_T_ between methadone and buprenorphine or a relationship between the prescribed dose of either substitute with *V*_T_, but the study was not sufficiently powered for such analyses.

### Opiate dependence: α1 and α5

**Fig. 3:**
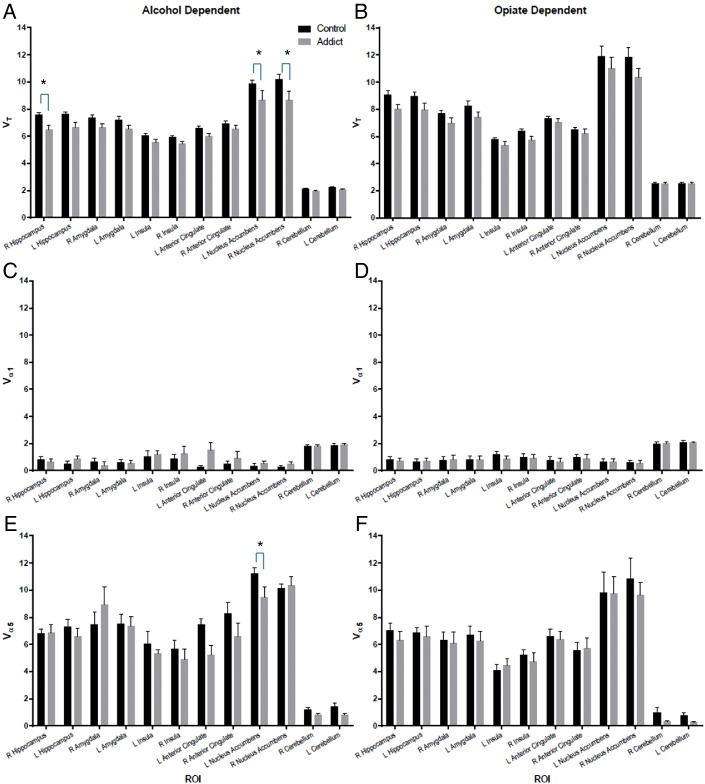
Comparison of [^11^C]Ro15 4513 *V*_T_, *V*_α__1_ and *V*_α__5_ (mean ± SEM) between controls (for alcohol: *n* = 11 (see Supplementary information for more details); for opiate *n* = 13) and alcohol (*n* = 8; A, C, E) and opiate (*n* = 12; B, D, F)-dependent individuals in selected regions of interest (ROIs). *t = 3.713; *p* < 0.01.

There was no effect of group on the α1 component (*F*(1, 1743) = 0.1020; *p* = 0.7495), and no individual regions were prominent after correction for multiple comparisons. There was, however, an effect of group on the α5 component (*F*(1, 1660) = 4.760; *p* = 0.0293) with lower α5 levels in the opiate-dependent group. The large inter-subject variability resulted in no significant differences being found in any individual ROI after correction for multiple comparisons (see Fig. 3B,D,F). No significant correlations with memory performance were found (see Fig. 2C,F,I).

## Discussion

In this first [^11^C]Ro15 4513 PET study of opiate dependence, we have shown significantly reduced levels of [^11^C]Ro15 4513 *V*_T_ in individuals on opiate substitute medication compared with controls. A region of interest analysis revealed a significant reduction in the right nucleus accumbens. This complements our study in abstinent alcohol dependence where we also reported reduced [^11^C]Ro15 4513 *V*_T_ in the nucleus accumbens. Using our spectral analytical approach to determine contribution of α1 and α5 binding to [^11^C]Ro15 4513 *V*_T_, we found lower levels of α5 subtype in nucleus accumbens in both alcohol and opiate dependence compared with controls no significant contribution from α1. Given the different neuropharmacology of opiates and alcohol, our studies support our original proposal that low α5 levels may be fundamental to addiction rather than only associated with alcoholism.

We are not aware of any other in vivo imaging study of the GABA-benzodiazepine receptor in opiate dependence in man. In alcoholism using non-selective tracers, [^11^C]flumazenil with PET or [^123^I]iomazenil with SPET, have consistently shown a reduction in GABA-benzodiazepine receptors particularly in the frontal cortex (Abi-Dargham et al., 1998, Lingford-Hughes et al., 1998) whilst with [^11^C]Ro15 4513, reductions are seen in limbic areas where binding is high (Lingford-Hughes et al., 2002, Lingford-Hughes et al., 2012). It would be interesting to know if a similar difference in distribution of [^11^C]flumazenil vs [^11^C]Ro15 4513 is seen in opiate dependence and the impact of the different opiate substitute medications. Whilst we did not see any differences between methadone and buprenorphine and [^11^C]Ro15 4513 binding, due to the small numbers we cannot exclude an interaction.

There are limited and inconsistent preclinical receptor binding studies to inform this clinical study. Direct competition between opiate substitute medication and [^11^C]Ro15 4513 is unlikely since opiate agonists have low affinity (~ μM) for the [^3^H]GABA site (Dingledine et al., 1978, Goldinger et al., 1981). Chronic morphine exposure has been reported to increase [^3^H]GABA binding (Ticku and Huffman, 1980) and to increase [^3^H]muscimol, [^3^H]flunitrazepam or [^3^H]Ro15 1788 (flumazenil) binding in the cortex, which return to control levels in withdrawal (Sivam et al., 1982, Lopez et al., 1990, Rocha et al., 1993). However, other studies using active and passive or acute and chronic morphine administration resulted in reduced or no changes in [^3^H]flunitrazepam binding in a range of cortical regions and hippocampus, although chronic morphine did reduce sensitivity to GABA-enhancement of binding (Smith et al., 1984, Sivam and Ho, 1982). Given the variability of models, length of opiate exposure and ligands for benzodiazepine receptor or GABA site markers used, it is difficult to extrapolate these findings to our clinical [^11^C]Ro15 4513 PET study. The clinical importance of the interaction between opiates and the GABA-ergic system suggests further characterisation would be beneficial. In addition, it would provide evidence to understand if the lower level of [^11^C]Ro15 4513 binding in alcohol and opiate addiction reflect a vulnerability endophenotype to addiction.

We have previously reported in our healthy control group that a current or previous history of smoking was associated with higher levels of [^11^C]Ro15 4513 binding compared with those who had not smoked; current smokers had lowest [^11^C]Ro15 4513 binding levels and were more similar to controls than ex-smokers (Stokes et al., 2014). Since all the opiate-dependent participants were current smokers, we are unable to distinguish between the roles of opiate and smoking dependence to [^11^C]Ro15 4513 binding. In the alcohol-dependent group, smokers had higher levels than the non-smokers, although there was only one ex-smoker and two smokers. An [^123^I]iomazenil SPET study reported higher binding in a non-smoker alcohol-dependent group compared with a smoker alcohol-dependent group after one week of abstinence but no differences were seen at four weeks (Staley et al., 2005). Given the differences in length of abstinence from alcohol, it is hard to directly compare the studies; however, both suggest that smoking tobacco may alter GABA-benzodiazepine receptor availability. If a reduction in α5 is critical in addiction, then why is tobacco smoking not associated with reduced [^11^C]Ro15 4513 binding? Analogous to studying alcohol dependence, where controls drink low amounts of alcohol, comparing non-smokers, non-dependent ‘chippers’ and dependent smokers will be required to determine impact of smoking versus addiction on [^11^C]Ro15 4513 binding.

Characterising benzodiazepine subtypes in man is important to inform our understanding about its involvement in neuropsychiatric disorders. The α1 subtype is ubiquitous (~ 60% of all benzodiazepine receptors), is found in the synapse and is responsible for fast inhibitory neurotransmission, whereas α5 is less abundant (~ 5%), extrasynaptic, and responsible for tonic inhibition (Rudolph and Knoflach, 2011). Each subtype is associated with particular roles such as sedation and anti-convulsant effects for the α1 subtype and mediation of alcohol reward and memory for α5. Using our spectral analytical approach, we have demonstrated that the lower [^11^C]Ro15 4513 *V*_T_ in the nucleus accumbens predominantly reflects lower α5 levels in alcohol and opiate dependence. There are no preclinical studies of α1 and α5 subtypes in opiate exposure. Whilst reduced α1 subtype protein and mRNA have been reported consistently from chronic alcohol exposure in male rats, by contrast increases or no change in binding of the α1 selective tracer, [^3^H]zolpidem, have been reported (Grobin et al., 2000). Similarly, alcohol-induced increases, decreases or no change have been reported in α5 subunit protein or mRNA in the cortex (Devaud et al., 1995, Charlton et al., 1997). It is difficult to extrapolate such preclinical studies to alcohol dependence in man with its greater complexity of alcohol exposure and withdrawal.

In alcohol dependence, we previously reported that [^11^C]Ro15 4513 *V*_T_ was significant lower in the hippocampus compared with healthy controls and that there was a positive relationship with delayed verbal memory performance (Lingford-Hughes et al., 2012). We further show here that this relationship between delayed verbal memory performance is associated with [^11^C]Ro15 4513 *V*_α__5_ and not *V*_α__1_ in the hippocampus in alcohol dependence. No such relationship was found in opiate dependence or either control group. This is consistent with our suggestion that hippocampal α5 levels here reflect adaptation to excessive alcohol exposure (Lingford-Hughes et al., 2012) since none in opiate group were alcohol dependent or had significant history of alcohol abuse. Therefore, we believe our confirmation of α5 rather than α1 being associated with memory adds to the growing evidence of the importance of α5 in memory in neuropsychiatric disorders in man (Atack, 2011). Concerning the role of the α5 subtype in reward, whilst there is preclinical evidence of lower α5 levels associated with reduced alcohol preference and alcohol-seeking (June et al., 2001), there are no comparable studies in opiate reward or dependence.

Whilst this is the first study of [^11^C]Ro15 4513 in opiate dependence, and we report for the first time delineation of α1 and α5 in both alcohol and opiate dependence, a limitation of our studies is that the numbers are small. As a consequence of the loss of the scanner on which the alcohol dependent and their control group were studied, we had to perform the study in opiate-dependent participants on a different scanner and acquire an additional control group. Therefore, any inferences from comparisons between alcohol and opiate dependence are cautiously stated. As with our alcohol dependence study (Lingford-Hughes et al., 2012), we think it unlikely that partial volume effects substantially contribute to the reductions in [^11^C]Ro15 4513 binding seen in the opiate-dependent group. We did not find any differences in striatal volume between groups and have previously reported no atrophy in the striatum in a similar group of opiate-dependent individuals (Reid et al., 2008).

In summary, we have shown lower [^11^C]Ro15 4513 binding associated with lower [^11^C]Ro15 4513 *V*_α__5_ in opiate dependence, particularly in the nucleus accumbens. We have also shown lower α5 levels in the nucleus accumbens in alcohol dependence, and suggest that such a reduction is fundamental to addiction and independent of the substance of abuse. We propose that lower levels of α5 could result in less GABA-ergic inhibitory activity on the mesolimbic dopaminergic system resulting in its greater instability and thus greater response to rewarding stimuli or salient cues. It remains to be elucidated whether these lower levels contribute to a vulnerability endophenotype for addiction more generally or result from substance abuse. Modulating the α5 subtype presents an innovative target to probe substance abuse and how it can be prevented or treated, particularly now agents selective for this receptor subtype are being developed for human use (Atack, 2011).

## Figures and Tables

**Table 1:** Demographic and clinical data.

Clinical variable	Control (opiate)	Opiate dependent
Number	13	12
Age	40 ± 5	36.2 ± 7.6
Beck's depression inventory	n/d	9.8 ± 7.9
Spielberger—State anxiety	n/d	33.64 ± 7.9
Spielberger—Trait anxiety	n/d	37.1 ± 9.8
Severity of Alcohol Dependence Questionnaire	n/a	n/a
Wechsler delayed verbal memory score	n/d	10.2 ± 3.4
Length of abstinence (months)	n/a	n/a
Years of opiate use or alcohol dependence	n/a	16 ± 6.8
Adjective checklist (opiate) withdrawal	n/a	4 ± 3.8
Adjective checklist (opiate) agonist-like	n/a	19.8 ± 3.9

n/a: not applicable; n/d: not done in whole sample.
