# Supplementary material for: Using [11C]Ro15 4513 PET to characterise GABA-benzodiazepine receptors in opiate addiction: Similarities and differences with alcoholism
Source: Neuroimage. 2016 May 15;132:1–7. doi: 10.1016/j.neuroimage.2016.02.005 (PMC4862962; doi:10.1016/j.neuroimage.2016.02.005)
Supplement: Supplementary file 1 — Supplementary material [file mmc1.docx]

Supplementary material to Lingford-Hughes et al

[^11^C]Ro15 4513 binding in opiate addiction: similarities and differences with alcoholism.

Materials and Methods.

Participants.

The abstinent (>6 weeks) alcohol dependent and its control group were included here for further analysis of their estimated [^11^C]Ro15 4513 specific distribution volumes (Myers et al., 2012). They have been described previously (18) but briefly, the average length of abstinence was 33.4 months (+44.7, range 2–108 months), with 14.9 + 5.3 years of alcohol dependence. In the alcohol dependent and control groups, recreational or occasional use of illicit drugs (e.g. cannabis, ecstasy, amphetamine), but not dependency (DSM-IV), was admissible. In the alcohol dependent group, 2 were smokers, 5 non-smokers and 1 ex-smoker and in its control group, 2 were current smokers, 3 ex-smokers and 6 non-smokers.

Individuals with clinical evidence of hepatic, cognitive or neurological impairment or medical disorder were excluded. No individual had a previous or current history of psychosis and none were taking psychotropic drugs.

The studies on opiate and alcohol dependence were performed with different PET scanners, consequently we cannot directly compare them and each required separate control groups.

*Clinical assessments.*

The alcohol dependent and their control group underwent a similar assessment to that completed by the opiate dependent and their control group and was described in the previous publication (Lingford-Hughes et al., 2012). It included the Beck Depression Inventory (Beck et al., 1961), Spielberger State - Trait Anxiety Inventory (Spielberger, 1970) and the Wechsler Memory Scale and logical memory with immediate and 30-minute delayed recall of two stories.

*[^11^C]Ro15 4513 PET*

We followed the same scanning protocol as described in Lingford-Hughes et al., (2002). [^11^C]Ro15 4513 was synthesized by *N*-methylation of the corresponding *N*-desmethyl derivative with [^11^C]iodomethane. The product was purified by reverse phase HPLC and the specific radioactivity was ~14000 MBq/μmol at end of synthesis.

[^11^C]Ro15 4513 scans of alcohol-dependent individuals and corresponding control subjects were performed using an ECAT EXACT3D HR++ PET scanner (CTI/Siemens, Knoxville, TN, USA) with performance characteristics as described previously (Lingford-Hughes et al., 2012), with prior 10-minute transmission scans for subsequent attenuation and scatter correction. A bolus injection of [^11^C]Ro15 4513 (mean ~370 MBq) in ~2ml was administered through an intravenous cannula sited in the dominant antecubital fossa vein. 20 dynamic frames (1 x 30, 4 x 15, 4 x 60, 2 x 150, 2 x 300, 7 x 600 s) of data were acquired in 3D mode over 90 minutes and reconstructed into images containing 78 contiguous transaxial slices.

Blood sampling was carried out to produce a calibrated, metabolite corrected plasma input function for quantification of distribution volume with spectral analysis (Lingford-Hughes et al., 2012).

***Image processing***

This was done using the same methods and approach as described for the opiate dependent and their control group except for the following aspects.

For the alcohol-dependent cohort, an [^11^C]Ro15 4513 PET template was created for accurate definition of ROIs due to lack of MR scans (Lingford-Hughes et al., 2012). Individual add-images were coregistered to individual MRI data, normalised to standard MNI space.

**Results.**

**Alcohol dependence: α1 and α5**

The lower [^11^C]Ro15 4513 V_T_ in the alcohol dependent group compared with controls previously reported (18) appeared to be driven by the slower α5 spectral component. There was no effect of Group on estimated V_α1_ (F (1, 876) = 1.483; p = 0.2237), but a significantly lower V_α5_ (F (1, 876) = 58.54; p < 0.0001). After correcting for multiple comparisons, the V_α5_ in the left nucleus accumbens remained significantly lower in the alcohol dependent group (t = 3.713; p < 0.01; see Figure 3A,C,E). A positive significant (p<0.005) correlation was found between hippocampal [^11^C]Ro15 4513 V_T_ with memory performance in alcohol dependent individuals. The relationship was driven by [^11^C]Ro15 4513 V_α5_ and not V_α1_, although did not reach statistical significance (see Figure 2B,E,H).

**Supplementary Table.**

Demographic and clinical data.

| Clinical Variable | Control  (alcohol) | Alcohol dependent |
| --- | --- | --- |
| number | 11 | 8 |
| Age | 43 + 7 | 44 + 7 |
| Beck’s depression inventory | 4.5 + 4.4 | 10.9 + 10.5 |
| Spielberger – State anxiety | 27.7 + 7.2 | 35.5 + 13.5 |
| Spielberger – Trait anxiety | 33.5 + 11.6 | 37.25 + 11.8 |
| Severity of Alcohol Dependence Questionnaire | 2 + 2 | 36 + 18 |
| Wechsler delayed verbal memory score | 13.2 + 2.7 | 10.1 + 4.9 |
| Length of abstinence (months) | n/a | 3.1 + 3.9 |
| Years of opiate use or alcohol dependence | n/a | 14.9 + 5.3 |
| Adjective checklist (opiate) withdrawal | n/a | n/a |
| Adjective checklist (opiate) agonist-like | n/a | n/a |

n/a: not applicable
